# Stress Response System in the Fish Skin—Welfare Measures Revisited

**DOI:** 10.3389/fphys.2019.00072

**Published:** 2019-02-08

**Authors:** Ewa Kulczykowska

**Affiliations:** Institute of Oceanology of the Polish Academy of Sciences, Sopot, Poland

**Keywords:** fish, welfare, skin, stress, melatonin, mucus, aquaculture, AFMK

## Abstract

The skin of vertebrates acts as a biological barrier defending the organism against many harmful environmental factors. It is well established that the main stress hormone cortisol, together with antioxidants such as melatonin (Mel) and its biologically active metabolites set up a local stress response system in the mammalian skin. Recently, our research group has shown that in fish there are basic conditions for the functioning of a cutaneous stress response system (CSRS) similar to that in mammals, where Mel with its biologically active metabolite AFMK (N1-acetyl-N2-formyl-5-methoxykynuramine) and cortisol act together to protect organism against unfavorable environment. Since aquaculture is making an increasing contribution to the global economy and new laws are demanding people to respect the welfare requirements of animals there has been increasing interest in indicators of fish well-being in aquaculture. This article addresses the problem of on-farm assessment of fish welfare and proposes the CSRS as a new source of information on the welfare status of farmed fish.

There has been growing public concern for the well-being of farmed fish since aquaculture is making an increasing contribution to the global economy and, at the same time, new laws are demanding people to respect the welfare requirements of animals to ensure their right treatment (for review, see Browman et al., 2018). This has resulted in considerable research on the impact of various aspects of aquaculture practice on fish welfare and in a growing body of literature identifying potentially harmful aspects thereof and proposing different approaches to assessing animal well-being (for review, see Huntingford et al., 2006; Ashley, 2007; Martins et al., 2012). The issue of the welfare of aquatic animals was revisited quite recently by Browman et al. (2018). Nowadays, there are many physiological, biochemical, and behavioral indicators of fish welfare, many of them linked with the systemic response to stress (see Reviews cited above). However, appropriate combinations of welfare measures feasible in a fish farm are still much sought-after. In this context, our research group of marine physiologists at the Institute of Oceanology of the Polish Academy of Sciences has recently begun to investigate the cutaneous system of response to stress (CSRS) as a new source of information on the welfare status of farmed fish.

The skin of vertebrates is more than just a protective barrier between the external and internal environments. This largest body organ, which is directly exposed to multiple external stressors, can initiate appropriate responses to preserve body homeostasis by generating signals for the systemic defense system, but not only. In, Slominski et al. (1995) proposed that there is a local system of response to stress in mammalian skin which is an equivalent to the hypothalamic-pituitary-adrenal axis (HPA). Since then, there has been growing evidence supporting that view and many biologically active molecules engaged in the system have been identified (for review, see Slominski et al., 2008, 2012). It turns out that there are not only stress hormones related to the HPA, such as corticotropin-releasing hormone, adrenocorticotropic hormone, and cortisol, but also antioxidants, e.g., melatonin (Mel), with its biologically active metabolites, the kuramines N1-acetyl- N2-formyl-5-methoxykynuramine (AFMK) and N1-acetyl-5-methoxykynuramine (AMK) (Slominski et al., 2002, 2005, 2012), all engaged in the inactivation of free radicals in the cells (Hardeland, 2008; Galano et al., 2013). However, the epidermis that constitutes the outer layer of skin in vertebrates is diverse. The formation of a stratum corneum in the process whereby living keratinocytes are transformed into non-living corneocytes making a barrier against water-loss, mechanical and chemical stress, and infection is essential for all terrestrial vertebrates (Alibardi, 2009). The process of keratinization occurs, though to a lesser extent, also in fish (Mittal and Banerjee, 1980). But in fish, the epithelial mucus layer constitutes a first line of defense (Shephard, 1994), and therefore we consider the mucus a part of fish CSRS (see below).

Since it has been demonstrated that the exposure of mammalian skin to numerous pathological agents and harmful environmental factors induces production of biologically active compounds acting at both the cutaneous and systemic level to protect homeostasis (Slominski et al., 2005, 2012), one might expect to find a similar mechanism in fish. Recently, our research group has shown that in fish there are basic conditions for the functioning of a CSRS similar to that in mammals. Indeed, the main stress hormone cortisol, and antioxidants Mel and AFMK are present in fish skin (Kulczykowska et al., 2018). Moreover, *in vitro* experiments with European flounder skin explants have demonstrated that cortisol added to the incubation medium can stimulate Mel and AFMK release in a dose-dependent manner (Kulczykowska et al., 2018). Furthermore, the concentrations of cortisol used in the experiments mimicked plasma cortisol levels in fish exposed to different types of stress, such as handling, confinement, high stock density or food-deprivation (Mommsen et al., 1999; Barton, 2002). A question arises as to whether Mel and AFMK are formed in fish skin. One can expect the answer to be “yes” because a constitutive expression of genes encoding aralkylamine N-acetyltransferase (AANAT; EC2.3.1.87; the enzyme in Mel biosynthesis), albeit low, has been found in the skin of both the rainbow trout (*Oncorhynchus mykiss*) (Fernández-Durán et al., 2007) and the three-spined stickleback (*Gasterosteus aculeatus*) (Kulczykowska et al., 2017). Moreover, specifically *cutaneous* synthesis of Mel would seem to be confirmed by studies in various fish species where higher Mel concentrations were found in their skin than in plasma or muscles (Kulczykowska et al., 2018; our unpublished data). It can thus be presumed that, in fish subjected to unfavorable conditions, when their circulating cortisol concentration is high, the cutaneous synthesis of Mel/AFMK will increase, even though AANAT gene expression and Mel/AFMK production in the skin of non-stressed individuals is normally low (discussed by Kulczykowska et al., 2018). A stimulation of Mel production in organs such as the gut and skin when exposed directly to environmental pollutants is considered a protective action in any vertebrate organism (Hardeland, 2005; Tan et al., 2007). For instance, in white stork nestlings (*Ciconia ciconia*) living near a copper smelter, toxic compounds in the diet can stimulate Mel synthesis on-the-spot in the gut, to serve as a first-line defensive step in the protective mechanism of the organism (Kulczykowska et al., 2007). Various oxidative stress measures are commonly used in fish biology (Birnie-Gauvin et al., 2017), but not yet Mel and its metabolites.

Certainly, one might expect to find some dissimilarity between the stress response systems in fish skin and in terrestrial vertebrates’ skin. The aquatic environment and the specific structure/function of fish skin make a difference! The skin in fish is covered by mucus and this outermost layer has been considered a first line of defense against a wide range of detrimental environmental conditions (Shephard, 1994) and pathogens (Benhamed et al., 2014). Analysis of skin mucus, which is continuously secreted by cells, contains many components, and is easily collected, can be a valuable source of information on the welfare status of farmed fish. Metabolites such as glucose, lactate, protein, and cortisol in skin mucus have already been studied to judge their suitability for determination of physiological response to different stressors (De Mercado et al., 2018; Fernández-Alacid et al., 2018). A positive relation between stress markers in plasma and skin mucus was recently demonstrated by Fernández-Alacid et al. (2019). This is good news from the point of view of elaboration of a new non-invasive, quick, and simple assay to detect stress responses in fish, especially in aquaculture. The current research of our group at the Institute of Oceanology focuses on the CSRS and its components, Mel and AFMK, synthesized and released in response to stress in fish. Analysis of cortisol together with Mel and AFMK in the mucus seems to be a promising approach to on-farm assessment of fish welfare.

## Author Contributions

The author confirms being the sole contributor of this work and has approved it for publication.

## Conflict of Interest Statement

The author declares that the research was conducted in the absence of any commercial or financial relationships that could be construed as a potential conflict of interest.
